# Bilateral Symptomatic Mucoid Degeneration of the Anterior Cruciate Ligament with Anterior Knee Pain but No Limited Knee Flexion

**DOI:** 10.1155/2021/5879121

**Published:** 2021-10-21

**Authors:** Koshiro Shimasaki, Tomokazu Yoshioka, Akihiro Kanamori, Masashi Yamazaki

**Affiliations:** ^1^Department of Orthopedic Surgery, Faculty of University of Tsukuba, 1-1-1 Tennodai, Tsukuba, Ibaraki 305-8575, Japan; ^2^Division of Regenerative Medicine for Musculoskeletal System, Faculty of Medicine, University of Tsukuba, 1-1-1 Tennodai, Tsukuba, Ibaraki 305-8575, Japan

## Abstract

Mucoid degeneration of the anterior cruciate ligament (ACL) is a rare cause of anterior knee pain (AKP). Some case reports have been published; however, it is difficult to diagnose and is often underdiagnosed or misdiagnosed because of its pathophysiological ambiguity. We report a rare case of a patient diagnosed with bilateral mucoid degeneration of the ACL with AKP and no limited joint range of motion (ROM). A 59-year-old man with spontaneous right AKP was admitted to our hospital. He first underwent arthroscopic resection of the thickened medial plica protruding far into the medial patellofemoral joint (PFJ) but felt little effectiveness thereafter. He then had an arthroscopic release of the lateral patellar retinaculum because of valgus knee and patellar instability, which resulted in only temporary improvement. Then, the AKP relapsed, this time with limitations in the ROM. Magnetic resonance imaging (MRI0 showed a diffuse, thickened ACL with a high inhomogeneous intensity in the T2-weighted and proton density weighted images and which looked similar to a celery stalk. Based on the patient's history and MRI findings, we suspected mucoid degeneration of the ACL and subsequently performed arthroscopic excision. At the same time, AKP appeared on the other side. Since the MRI demonstrated a similar celery stalk image as before, the same operation was performed on this side, as well. Finally, AKP and the limitation of the ROM were relieved approximately one month after surgery. Due to the patient only suffering from AKP with a preserved ROM, it took about 14 months to diagnose this disease. It should, therefore, always be considered in cases of AKP alone.

## 1. Introduction

Mucoid degeneration of the ACL is a rare disease in the middle-aged patient group without a history of any major knee trauma, as first reported by Kumar et al. in 1999 [1]. Some case reports show that most patients experience spontaneous knee pain with limitations in joint range of motion (ROM) due to the displacement of a large proportion of mucoid tissue in the ACL; however, its pathophysiology remains unclear.

We report a very rare case of a patient who had been diagnosed with bilateral mucoid degeneration of the ACL, presenting with AKP and no limited joint ROM.

The purpose of this article is to review the prior literature and describe some features of clinical examination, imaging, and arthroscopy to better characterize this ambiguous entity.

## 2. Case Presentation

**Figure 1:**
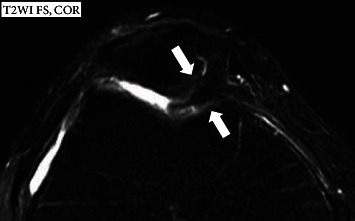
MRI showed a thickening of the medial plica protruding far into the medial patellofemoral joint (PFJ).

A 59-year-old male who had no prior significant trauma was admitted to our hospital for evaluation of a right AKP of two weeks. There was little swelling without limitations in the knee joint's ROM nor instability. There was, however, tenderness at the medial joint line of the knee. A plane X-ray showed a slight degenerative change on the medial side of the knee. As MRI showed a thickening of the medial plica protruding far into the medial patellofemoral joint (PFJ) (Figure 1), we performed arthroscopic resection; however, there was little improvement.

**Figure 2:**
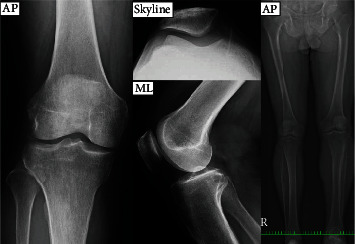
Valgus knee and patellar instability appeared on a plane X-ray with a large Q angle: 17°, tilt angle: 21°, and lateral shift: 18%.

Afterwards, valgus knee and patellar instability appeared on a plane X-ray with a large Q angle: 17°, tilt angle: 21°, and lateral shift: 18% (Figure 2). The patient subsequently underwent release of the lateral patellar retinaculum. However, not only was the result the same as the prior surgery, but the patient began experiencing limitations in his knee joint's ROM (15-90°) as well as AKP on the other side.

**Figure 3:**
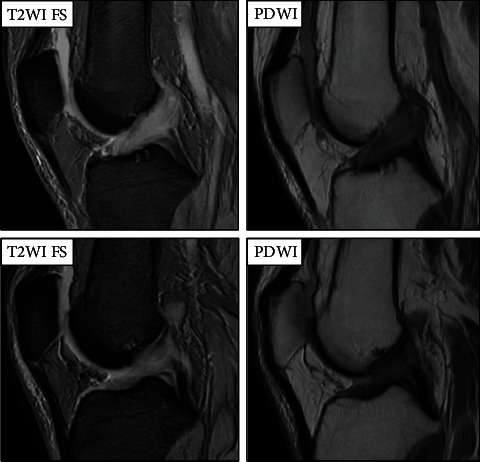
MRI showed diffuse thickening of both ACLs, which are called celery stalk sign, with high inhomogeneous intensity in the T2-weighted and proton density weighted images.

**Figure 4:**
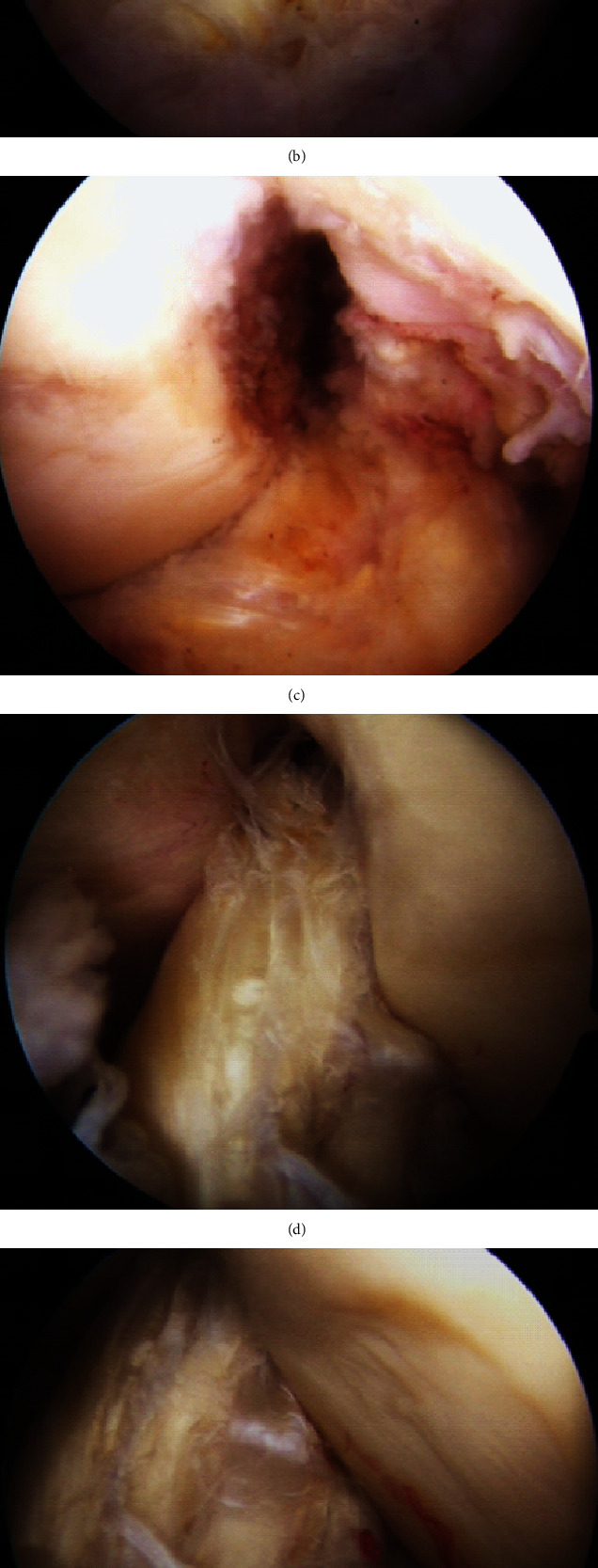
Arthroscopy showed (a, d) hypertrophied ACLs and the (b, d) presence of intraligamentous mucoid degeneration. (e) The posterolateral bundles in the ACLs were slightly loose at the lateral portion of the intercondylar notch. (c, e) Further, there was a partial depression of cartilage in the same region.

MRI showed diffuse thickening of both ACLs with high inhomogeneous intensity in the T2-weighted and proton density weighted images (Figure 3). Based on the patient's history and MRI findings, we suspected bilateral mucoid degeneration of the ACL and performed an arthroscopy. Upon examination, we found hypertrophied ACLs and the presence of intraligamentous mucoid degeneration. The posterolateral bundles in the ACLs were slightly loose at the lateral portion of the intercondylar notch. Further, there was a partial depression of cartilage in the same region (Figure 4).

**Figure 5:**
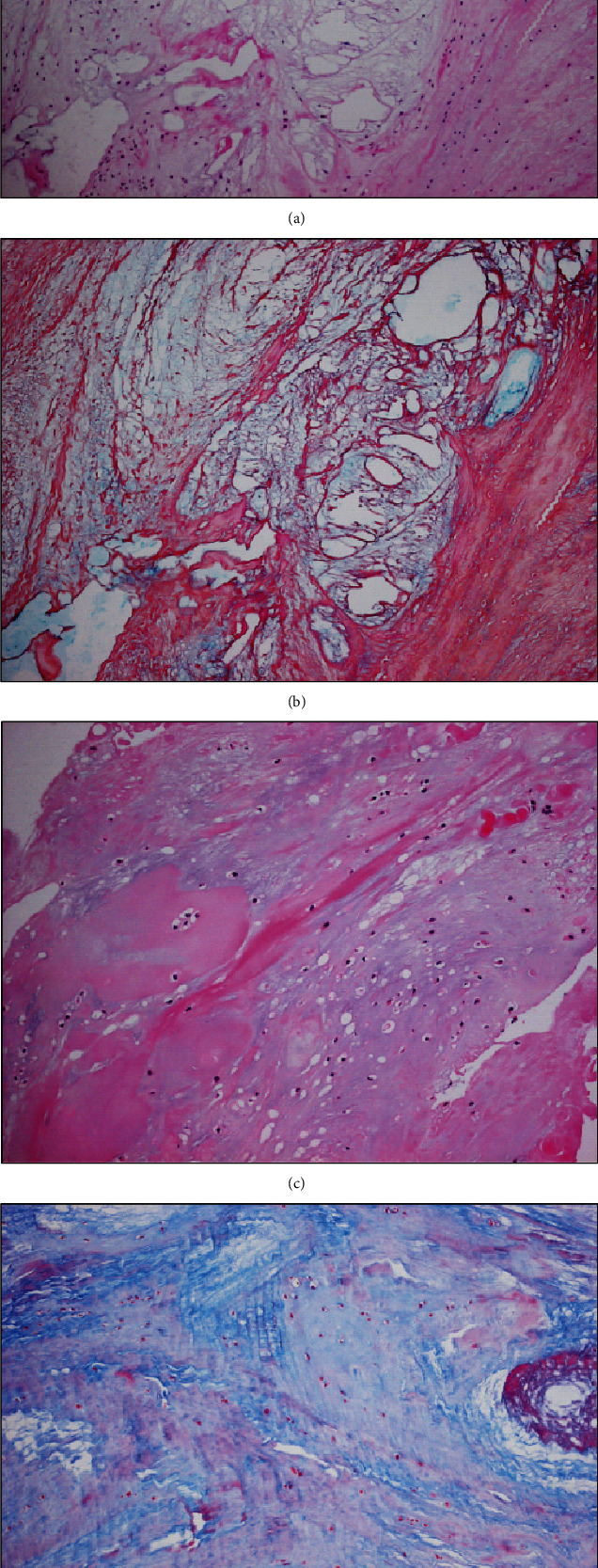
(a, c) Both hematoxylin and eosin stain, (b) Alcian blue stain, and (d) Masson trichrome stain. All of these results indicated the presence of mucoid degeneration.

To support our interventions, pathological results also indicated the presence of mucoid degeneration in the ACLs (Figure 5). We debrided only the right ACL completely at this time. Two months after the surgery, both AKP and the limitation of the ROM on the right side improved greatly. In contrast, conservative treatment on the other side failed, so we performed the same operation as with the first.

Postoperatively, the AKP disappeared, and the patient regained the full ROM. Total work incapacity lasted 4 weeks after the final operation. During the follow-up visit, which occurred half a year postoperatively, the patient reported no pain, instability, nor restrictions in activities of daily living.

## 3. Discussion

Mucoid degeneration of the ACL, with a prevalence rate of approximately 0.4%, is a rare cause of knee pain in the middle-aged patient group without a history of any major knee trauma [2]. It is said that knee pain with a limitation of joint ROM is one of the most common symptoms; however, many cases are found incidentally without contributory symptoms.

Although the etiology and onset mechanism of this disease remain unclear, some case reports suggested that multiple factors in the anatomical structure of the knee might be involved in this disease, for example, (1) the degenerative change in the medial side of the knee [3], (2) anterior translation of the tibia caused by a large posterior tibial slope [4], and (3) the narrowness of intercondylar notch [5], which can cause a repetitive and microtraumatic disruption of ligament fibres.

The main and useful method for diagnosis is MRI demonstrating intermediate signal in T1-weighted and increased signal in T2-weighted images diffusely within the ACL. “The celery stalk” sign is one of the most specific signals, in which low signals of longitudinal fibres are separated from each other by a higher-signal mucinous material, and is best appreciated on T2-weighted images [6]. It is estimated that only a few patients may be observed conservatively as a partial tear of the ACL because of its rarity. At arthroscopy, we can typically see homogeneous and hypertrophic ligaments with yellow soft tissue inside the mucoid degeneration of the ACL.

In our case, we found bulky ACLs and celery stalk signs on both sides via MRI (Figure 3) as well as bilateral diffuse yellowish degeneration and mucoid tissue of the ACL via arthroscopy. A part of the posterolateral bundle (PLB) of the ACL was not tensioned and damaged at the lateral portion of the intercondylar notch (Figure 4). Moreover, there was a partial depression of cartilage at the same portion, which suggested the presence of impingement between the ACL and lateral portion of the intercondylar notch. Pathologically, mucoid tissue existed among the ligament fibres and degeneration of microcysts on hematoxylin-eosin and Alcian blue staining (Figure 5), which led us to diagnose bilateral mucoid degeneration of the ACL.

In treatment, we have not yet established a standard method. Some prior reports stated that partial resection of the degeneration area results in a remarkable improvement in knee pain and joint ROM [7], while others have reported that total removal of the ACL [8] or PLB [9] could relieve painful symptoms. However, they also reported that instability in the knee joint often appeared 2-5 years after the partial resection and repair were conducted [10], and complete tear of the ACL occurred in about 10% of patients after the surgery [11]. We should aggressively consider reconstruction at the same time in patients who are young or active [7] or select PS-TKA in fear of mucoid degeneration of the posterior cruciate ligament [12].

In our case, we decided to opt for bilateral intervention, concerning mucoid degeneration in both ACLs, to relieve the patient's pain completely. We had already performed surgery several times, although knee instability after the operation was a continuous concern. We continued to carefully monitor his progress following surgery should further ACL repair be required.

In summary, patellar instability and patellofemoral disorder can be considered as different diagnoses, presenting AKP in addition to mucoid degeneration of the ACL. Although we show the important points for distinguishing these diseases in Table 1, the rareness of mucoid degeneration of the ACL makes it difficult to diagnose, especially on both sides without limitation of knee joint ROM in the initial stage like our patient. It is necessary to conduct wider investigations including physical findings, imaging, and sequential changes, for an accurate diagnosis. It is imperative to consider mucoid degeneration of the ACL as one of the diseases presenting AKP.

## Figures and Tables

**Table 1:** Differences among similar diseases.

	Mucoid degeneration	Plica syndrome	Patellar instability
Pain	Anterior, posterior	Anterior-medial	Anterior
Swelling	+	±	–
Limitation of ROM	+	+	+
Patella apprehension	–	–	+
Plane X-ray	No specific findings	No specific findings	Patella Alta Patella tilting angle ↑ Q angle ↑
MRI	Celery stalk sign	Thickening of plica	Damage in MPFL, cartilage
Arthroscopy	Thickening ACL Mucoid tissue in ACL	Plica protruding into medial PFJ	Damage in cartilage
